# Prevalence and severity of Anxiety and Depression among Primary caregivers with correlation to their Psychiatric outpatients diagnostic entity according to ICD-10

**DOI:** 10.1192/j.eurpsy.2022.494

**Published:** 2022-09-01

**Authors:** I. Afridi

**Affiliations:** CPSP (College of Physicians & Surgeons, Pakistan), Faculty Of Psychiatry,, Karachi, Pakistan

**Keywords:** Anxiety, Depression, Caregivers, Outpatients

## Abstract

**Introduction:**

The impact of psychiatric patients on their primary caregivers is important. Outcome of different psychiatric disorders may also depends open the mental health of their caregivers. Therefore assessment of the prevalence and severity of Anxiety and depressive disorders may help to improve the wellbeing the caregivers as well as their sufferers.

**Objectives:**

To assess the prevalence and severity of Anxiety and Depression among primary caregivers of psychiatric outpatients. To correlate the psychiatric diagnosis according to ICD-10, of the patients with their caregivers anxiety and depression.

**Methods:**

The study was carried out in private Psychiatric Hospital with primary caregivers of psychiatric outpatients. One hundred and eighty consecutive and consenting participants were selected. Besides, applying semi-structured preform, designed for this purpose, anxiety and depression levels of these individuals were assessed using the locally validated version of HOSPITAL ANXIETY AND DEPRESSION SCALE (HADS). For the diagnostic categorization ICD-10 of WHO was used. Data was tabulated and analyzed using SPPS 17 version.

**Results:**

Among the 180 caregivers, between the age range of 18 to 92 years, 44% were found to be suffering from anxiety, and 68.56% were suffering from depressive disorders. Anxiety and Depression (combined) existed among 74.85% of the caregivers of psychiatric outpatients. Frequency of depressive disorder was found higher among the brothers (19.08%) as caregivers, followed by mother (18.32%), relatives (16.03%), and in spouses (15.26%).

**Conclusions:**

Caregivers of psychiatric patients, especially females, are more vulnerable to anxiety and depression irrespective of the type of their patients’ illness.

**Disclosure:**

No significant relationships.

